# Partial visual loss disrupts the relationship between judged room size and sound source distance

**DOI:** 10.1007/s00221-021-06235-0

**Published:** 2021-10-08

**Authors:** Andrew J. Kolarik, Brian C. J. Moore, Silvia Cirstea, Rajiv Raman, Sarika Gopalakrishnan, Shahina Pardhan

**Affiliations:** 1 Vision and Eye Research Institute, Faculty of Health, Education, Medicine and Social Care, Anglia Ruskin University, Cambridge, UK; 2 Cambridge Hearing Group, Department of Psychology, University of Cambridge, Cambridge, UK; 3 School of Psychology, University of East Anglia, Norwich, UK; 4 School of Computing and Information Science, Anglia Ruskin University, Cambridge, UK; 5 Shri Bhagwan Mahavir Vitreoretinal Services, Sankara Nethralaya Eye Hospital, Chennai, India; 6 Faculty of Low Vision Care, Elite School of Optometry, Chennai, India; 7 Low Vision Care Department, Sankara Nethralaya Eye Hospital, Chennai, India

**Keywords:** Vision loss, Spatial hearing, Auditory distance, Multisensory plasticity, Sound localization

## Abstract

Visual spatial information plays an important role in calibrating auditory space. Blindness results in deficits in a number of auditory abilities, which have been explained in terms of the hypothesis that visual information is needed to calibrate audition. When judging the size of a novel room when only auditory cues are available, normally sighted participants may use the location of the farthest sound source to infer the nearest possible distance of the far wall. However, for people with partial visual loss (distinct from blindness in that some vision is present), such a strategy may not be reliable if vision is needed to calibrate auditory cues for distance. In the current study, participants were presented with sounds at different distances (ranging from 1.2 to 13.8 m) in a simulated reverberant (*T*_60_ = 700 ms) or anechoic room. Farthest distance judgments and room size judgments (volume and area) were obtained from blindfolded participants (18 normally sighted, 38 partially sighted) for speech, music, and noise stimuli. With sighted participants, the judged room volume and farthest sound source distance estimates were positively correlated (*p* < 0.05) for all conditions. Participants with visual losses showed no significant correlations for any of the conditions tested. A similar pattern of results was observed for the correlations between farthest distance and room floor area estimates. Results demonstrate that partial visual loss disrupts the relationship between judged room size and sound source distance that is shown by sighted participants.

## Introduction

Estimation of the three-dimensional characteristics of a room, such as its layout and its size, plays an important role in orientation (Loomis et al. 1993), path planning (Kolarik et al. 2016b, 2017c), and safe navigation (Hackney et al. 2014; Kolarik et al. 2016c). Acoustic information can be very useful for blind or partially sighted individuals to help estimate the size of a room. In the current paper, blindness is defined as full visual loss or light perception only, in which case no visual spatial information is available. Partial visual impairment (VI) is defined as remaining vision that is better than light perception and where visual spatial information is present but degraded. In principle, auditory information regarding the location of the most distant sound source allows inference of the nearest possible distance of the far wall, since large distances between the source and listener are only possible in large rooms (Calcagno et al. 2012). For blind or partially sighted individuals confronted by an unfamiliar room, these cues are likely to be useful when first entering the room, to allow creation of an initial internal spatial representation of its dimensions. Investigation of estimated room size and sound source distance provides a good way of addressing the effects of visual loss on auditory abilities, as it provides insight regarding the effects of vision loss on the internal representations of the external environment based on audition, and how vision (either intact or degraded) is used to calibrate auditory space. In addition, to date, there has been relatively little research investigating what strategies individuals with partial visual loss use to navigate spaces in their daily life, or whether they use the same strategies as employed by blind individuals. For example, it might be the case that blind individuals use the location of the farthest sound source to infer the nearest possible distance of the far wall to plan their navigation, whereas VI individuals may instead rely on the pattern of sound reflections within a room. Room size perception using audition is also under-researched in populations with full vision, as well as those with blindness or VI.

If there is a significant correlation between judged room size and judged farthest sound source distance in novel rooms, this would support the idea that the location of the farthest sound source is used to infer the nearest possible distance of the far wall. A relationship between farthest distance and room size judgments has been demonstrated for blindfolded normally sighted (participants with visual acuities of at least 6/6 in each eye) and fully blind participants (Kolarik et al. 2013d). However, the effects of VI are currently unknown. For individuals with usable vision, under normal conditions room size judgement is likely to be heavily dependent on visual information. For VI participants, use of the degraded visual signal to calibrate audition might affect auditory spatial judgments of room size. We investigated whether people with VI show the same type of association between judged distance of the farthest sound source and judged room size as sighted controls.

Blindness can result in large changes in auditory abilities, with impaired auditory abilities in certain tasks, improved abilities in others, and no change in other tasks (for reviews, see Collignon et al. (2009), Voss et al. (2010), Kolarik et al. (2016a, 2021) and Voss (2016)). For example, blind participants have been shown to display marked deficits for tasks involving absolute judgments (Kolarik et al. 2017a), spatial bisection (Gori et al. 2014), the localization of sounds in elevation (Zwiers et al. 2001; Lewald 2002), and perception of the location of a sound source in relation to external auditory landmarks (Vercillo et al. 2018). These poorer abilities have been accounted for in terms of the perceptual deficiency hypothesis, which suggests that an intact visual signal is required to accurately calibrate audition, without which auditory spatial performance is worse (Axelrod 1959; Jones 1975). However, other auditory abilities become enhanced following full blindness, including azimuth judgments (Lessard et al. 1998) underpinned by more effective use of spectral information (Doucet et al. 2005; Voss et al. 2011), distance discrimination (Voss et al. 2004; Kolarik et al. 2013b), performance of a minimum-audible-angle task (Voss et al. 2004), auditory spatial attention in peripheral space (Röder et al. 1999), frequency discrimination (Gougoux et al. 2004), and perception of auditory motion (Lewald 2013). Battal et al. (2020) showed that auditory discrimination abilities were enhanced in blind participants in the horizontal plane for peripheral areas, as well as in the vertical plane, in rear and frontal space. The enhanced performance following visual loss has been explained in terms of the perceptual enhancement hypothesis, which postulates that lack of an intact visual signal will lead to an increased reliance on utilizing the available auditory cues (Rice 1970), possibly due to sensory compensatory processes such as cortical reorganization (Voss and Zatorre 2012).

Similar to full blindness, people with VI show impaired auditory abilities for certain tasks, improved abilities for other tasks, and no change in others, although there are fewer studies that have examined the effect of VI on audition. Enhanced auditory localization in azimuth has been shown for participants with blindness in one eye only (Hoover et al. 2012). VI participants also self-reported better abilities than sighted participants in several auditory situations, including localizing the spatial position of the person talking during a conversation and being able to to follow speech that switches between several different talkers (Kolarik et al. 2017b). Individuals with other vision conditions also show different auditory performance compared to sighted controls. Myopic participants were reported to display significantly better azimuth localization abilities than sighted participants (Dufour and Gérard 2000; Després et al. 2005a), and myopic and amblyopic participants showed enhanced performance in a self-positioning task (Després et al. 2005b). VI children also performed significantly better than sighted children in a task involving locating the end point of a sound that was moving in the horizontal and vertical planes (Cappagli et al. 2017). Conversely, VI was associated with poorer auditory judgments of azimuth than for sighted controls (Lessard et al. 1998)*.* Participants with VI displayed a bias for perceiving the location of a sound as shifted towards the center of an array of loudspeakers (Ahmad et al. 2019) compared to sighted controls, and greater severity of visual impairment decreased the consistency of auditory distance judgments (Kolarik et al. 2020). Finally, other studies showed no effect of VI on auditory abilities in tasks requiring distance discrimination (Kolarik et al. 2013b) or indicating the spatial position of a single sound source on a vertical surface (Cappagli et al. 2017), when compared to sighted controls. Whether an auditory deficit or enhancement occurs appears to be task dependent, and factors that influence whether an auditory ability is enhanced or deteriorates in people with VI remain to be clarified (Kolarik et al. 2021).

In the current study, a normally sighted group and people with a range of severities of VI gave estimates of absolute distance and room size using auditory information alone. For normally sighted participants, vision provides important information in the calibration of auditory spatial cues, since visual estimates of distance are considerably more accurate than those for audition (Da Silva 1985; Loomis et al. 1998). It has been shown that short-term visual observation of the testing environment can be used to calibrate auditory spatial performance for azimuth in a reverberant room (Tonelli et al. 2015). Evidence that vision promotes auditory localization was shown in a study reporting that visual feedback resulted in lower absolute localization errors than when vision was unavailable (Tabry et al. 2013). Visual feedback and sensory-motor feedback (such as touching the sound source) are used to continually update internal representations of auditory space, so that auditory and visual spatial representations are aligned (Lewald 2013). For sighted participants, significant correlations exist between farthest distance judgments and room size judgments (Kolarik et al. 2013d). For VI participants with remaining usable vision, it is likely that although visual information is used to calibrate audition, the degraded visual cues may be unreliable or systematically in error when calibrating auditory spatial estimates. It was, thus, hypothesized that: (1) for sighted controls, calibration of audition using an intact visual signal would allow participants to rely on farthest distance estimates to judge room size, indicated by a significant correlation between farthest distance and room size judgments; (2) for VI participants, the degraded visual signal would lead to poorer calibration of auditory spatial cues, so that participants would not be able to rely on farthest distance estimates to judge room size and would, thus, show no correlation between judged room size and sound source distance. While Kolarik et al. (2013d) showed a significant correlation between farthest distance judgments and room size judgments for fully blind participants, the current experiment assessed whether VI participants performed differently due to the presence of remaining vision.

Anechoic space can be thought of as a room of infinite size, since the locations of the walls, floor and ceiling are not defined, and the signals at the ears of the participant are independent of the virtual room size. However, when visual information is not available, an anechoic room is probably not perceived as infinite in size. For a sound source of fixed intensity, the level at the listener’s ears decreases as the distance of the source increases, and the level cue may be used to estimate the distance of the source and hence to infer the nearest possible distance to the far wall. For sound sources of fixed intensity presented in anechoic rooms that are either real (Etchemendy et al. 2017) or simulated (Kolarik et al. 2013d), sighted participants’ auditory room size estimates increased with the distance of the farthest presented sound source, consistent with the idea of the level cue being used to estimate the nearest possible distance of the far wall. Hence, testing in simulated anechoic rooms provides useful baseline data for investigating the relationship between judged distance of the farthest sound sources and judged room size.

We tested the hypothesis that farthest auditory distance estimates and room size judgments (assessed using room volume, floor area, length, width and height estimates) were correlated for sighted participants but not for participants with any severity of VI. We also investigated whether the hypothesized effect of VI on the relationship between judged room size and farthest sound source distance generalized across different room environments and stimuli. Participants were tested in anechoic and reverberant rooms, using speech, music and noise stimuli. Participants made spatial judgments in simulated anechoic and reverberant rooms, to investigate whether the room reflections influenced the relationship between auditory distance estimates and room size judgments in participants with a range of severities of VI.

## Methods

The virtualization methods, stimuli tested, and procedures were similar to those used in our previous experiments that investigated auditory distance estimation (Kolarik et al. 2013c, 2017a) and room size estimation (Kolarik et al. 2013d) for sighted and fully blind (full visual loss or light perception only) groups. The stimuli are available from the authors upon reasonable request.

### Participants

56 Participants in total (18 normally sighted, 13 females, and 38 VI participants, 16 females) took part in the experiments. Participants were divided into groups based on their visual acuities, which were used as an indicator of the severity of their VI (Group 1: sighted controls, Group 2: mild VI, Group 3: mid-range VI, and Group 4: severe VI). The category boundaries were chosen so as to include participants with a wide range of visual losses, where the boundaries were approximately equivalent to WHO classifications of visual loss: mild (WHO categories 0–1, labeled as mild or moderate VI), mid-range (WHO 2–4, severe VI and blindness), and severe (WHO 4–5, blindness). Participants with mild VI were able to see light, colors and shapes, but vision might be unclear or hazy or areas of the visual field might be compromised, leading to difficulties in tasks such as recognizing street signs or faces. Individuals with mid-range VI had some useable form vision, shape recognition and the ability to perceive some movement. Those with severe VI had light perception only. The current category boundaries were chosen to distinguish different severities of VI (where usable vision is present) from total blindness. This is distinct from the WHO classification of “blindness”, where categories 3–5 are labeled as “blindness,” despite usable vision being available within this range. Sample sizes were dictated by the availability of the VI participants. Information about the groups is shown in Table 1, and details of the individual participants with VI are shown in Table 5 in the Appendix. The dataset in the current paper was the same as that used in the study by Kolarik et al. (2020), in which the correlation between visual acuity and judged room size was assessed. In the current study, the correlation between judged room size and judged sound source distance was assessed. The sighted controls had visual acuities of at least 6/6 in each eye. One-way ANOVAs showed that age did not differ significantly across groups (*p* > 0.05), and for the groups with VI, there was no significant difference in duration of VI (*p* > 0.05).

**Table 1:** Details of the participant groups

Group	*n*	LogMAR	Mean age (range), years
1. Normally sighted	18	0	21.1 (20–25)
2. Mild visual impairment	16	0.1–1	21.7 (18–31)
3. Mid-range visual impairment	12	1.1–2.9	21.1 (17–28)
4. Severe visual impairment	10	3–4	21.9 (18–31)

The number of participants (*n*), LogMAR [the Logarithm of the Minimum Angle of Resolution, a measure of visual acuity], mean age and range are given for each group. Group 1 were sighted controls. Groups 2, 3 and 4 had mild, mid-range, and severe VI, respectively

Previous studies of fully blind individuals have shown that there is a critical period (birth to approximately 14 years of age) for plasticity of the occipital cortex (Cohen et al. 1999), which has been linked to changes in auditory localization abilities (Collignon et al. 2009). Studies have previously classified blind participants as early- or late-onset groups, where early-onset is defined as 0–14 years of age (Gougoux et al. 2005; Voss et al. 2008). Following a recent study where the cut-off age was set to 6 years (Amadeo et al. 2019), Table 5 labels VI participants as early-onset (0–6 years of age) or late-onset VI (7 years of age or more). Thirty one VI participants were classified as early-onset and seven were classified as late-onset (five mild VI, and two mid-range VI).

Unfortunately, it was not possible to match the four groups for gender, due to the limited availability of VI participants. Effects of gender on auditory spatial processing have been reported in the literature, including left hemisphere differences in functional organization in the processing of monaural information (Lewald 2004). However, it is currently unknown whether gender plays a role in spatial judgements of distance and room size in the normally sighted and visually impaired populations, and this could be investigated in future work.

Participants were recruited from the Sankara Nethralaya Eye Hospital in Chennai, India, and audiograms for all participants were obtained using the procedures recommended by the British Society of Audiology (2011). Pure-tone-average better-ear hearing thresholds across the frequencies 0.5, 1, 2, 4, 6, and 8 kHz were less than or equal to 25 dB HL, indicating that all participants had normal or near-normal hearing. The nature and possible consequences of the testing were described to the participants, who then provided informed consent. The tenets of the Declaration of Helsinki were followed throughout testing. Ethical approval was provided by the Ethics Panels of Anglia Ruskin University and Sankara Nethralaya Eye Hospital.

### Apparatus and data acquisition

**Fig. 1:**
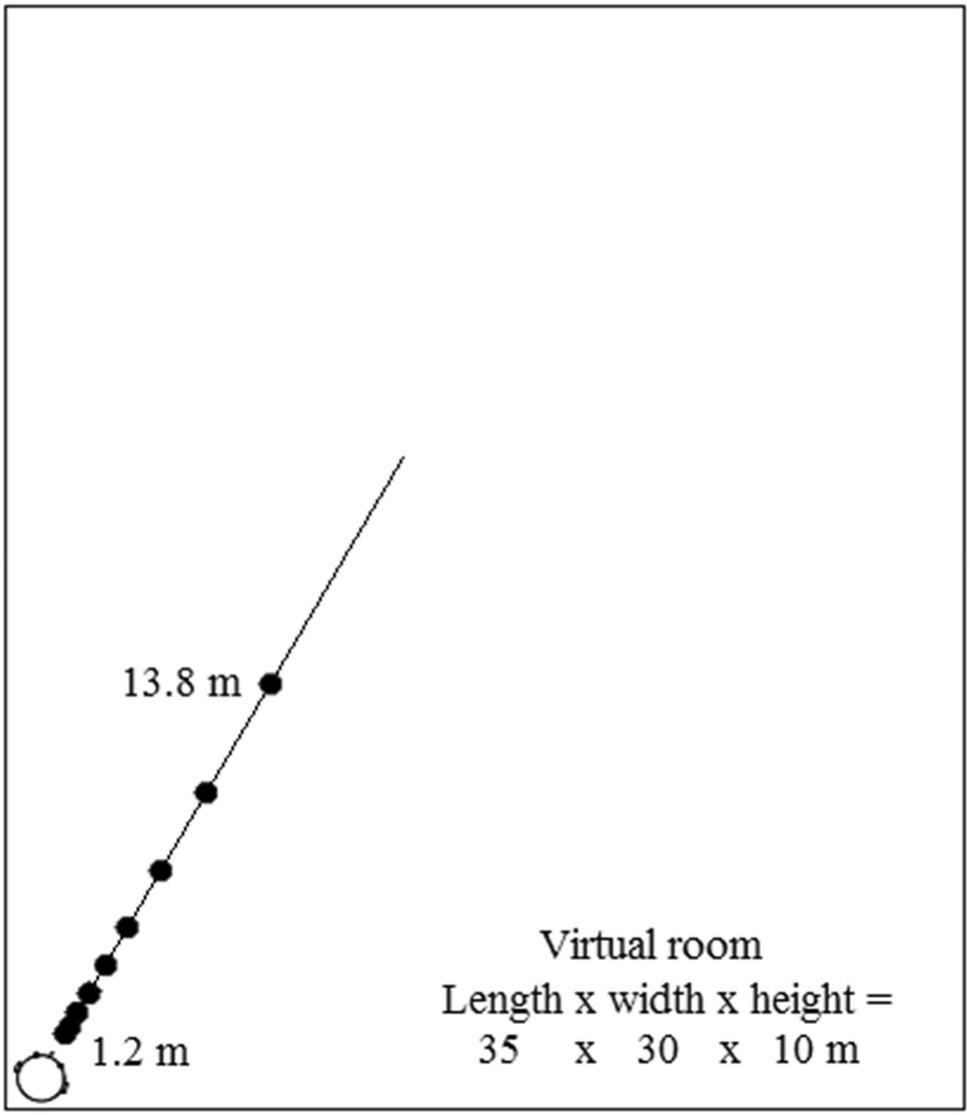
Layout of the virtual room. The position of the participant was simulated to be in the near-left corner. Closed circles show the positions of the sound sources, which were presented in front of the participant at virtual distances ranging from 1.2 to 13.8 m

Participants were tested in Sankara Nethralaya Eye Hospital in a room measuring 3.1 × 2.9 m, height 2.5 m, with an ambient sound level of approximately 39 dBA. A custom-written MATLAB script (Mathworks, Inc.) was used to produce the stimuli. Participant responses were recorded using a response interface on an Asus AA185 computer. The stimuli were produced using procedures detailed in our previous work (Kolarik et al. 2013a, b, c, d, 2017a), and were presented using Sennheiser HD219 headphones. An image-source model (Allen and Berkley 1979; Lehmann and Johansson 2008) was used to simulate an anechoic room or a reverberant room with dimensions 35 × 30 × 10 m (length × width × height). For the reverberant virtual room, the reverberation time *T*_60_ was set at 700 ms, matching the *T*_60_ used in previous work (Zahorik 2002b; Kolarik et al. 2017a). This *T*_60_ value is similar to that found in offices and living rooms (400–800 ms) and classrooms (400–1200 ms) (Nábĕlek and Nábĕlek 1994; Smaldino et al. 2008; Crukley et al. 2011). Using the image-source model, a room impulse response (RIR) was created between a virtual sound source and a receiver situated at a certain virtual distance from the source. Convolution of the RIR with a sound stimulus produced a simulation of the sound heard from a virtual distance within the room. The simulated sound sources, as shown in Fig. 1, were set at a virtual height of 1 m, at 0° elevation and 0° azimuth relative to the center of the head of the participant, who was located 1 m from each wall in the near-left corner facing forwards at 30° relative to the longer wall. These parameters were chosen to match those used in previous studies investigating the auditory perception of distance (Kolarik et al. 2013c, 2016a, 2017a).

The stimuli used were speech, music, and broadband white noise, chosen as they varied in their spectro-temporal characteristics (Kolarik et al. 2013d). The speech was composed of single sentences read by a male at a conversational level, taken at random from the Bench–Kowal–Bamford stimuli (Bench et al. 1979), and used in previous experiments (Akeroyd et al. 2007; Kolarik et al. 2013d, 2017a). The sentences were sampled at 22.05 Hz with 16-bit resolution and had a duration of 1.5 s. The music was a segment of a jazz trio (piano, bass and drums) sampled at 22.05 Hz with 16-bit resolution and with a duration of 7.3 s, as used in previous experiments (Moore et al. 2011; Kolarik et al. 2013d, 2017a). The white noise burst had a flat frequency spectrum at the source over the range 0.6–12 kHz. It was sampled at 44.1 kHz with 16-bit resolution and had a duration of 90 ms and rise/fall times of 10 ms, as used previously (Voss et al. 2004; Kolarik et al. 2013b, d, 2017a). The stimuli were chosen to match those used in previous studies, and thus differed in duration across conditions.

Sounds were simulated to originate from distances of 1.22, 1.72, 2.44, 3.45, 4.88, 6.90, 9.75 or 13.79 m from the participant, as used previously (Zahorik 2002a; Kolarik et al. 2013d, 2017a). All stimuli were generated prior to the experiment and accessed from saved files. The sound level at the position of the center of the participant’s head was 65 dB SPL (unweighted) for a virtual distance of 1 m. The simulated source level was fixed so the level at the participant’s head decreased with increasing simulated distance. Spatial rendering was performed by convolution of the direct sound component with non-individualized head-related transfer functions (HRTFs), using measurements made by Gardner and Martin (1995), as done previously (Voss et al. 2011; Kolarik et al. 2013b, d, 2017a). These measurements were obtained using a Knowles Electronics Manikin for Acoustics Research in an anechoic environment with a 1.4 m distance between the manikin and sound source. HRTFs are not dependent on distance when the sound source and receiver are more than 1 m apart (Otani et al. 2009), and HRTFs measured at 1 m can be used to simulate sounds located at greater distances (Brungart and Scott 2001). Although using non-individualized HRTFs can affect the extent to which sounds are perceived to be external to the head, recent work showed that using non-individualized HRTFs does not impair auditory distance perception (Prud'homme and Lavandier 2020). The stimulus generation technique used in the current study generates sounds for which the source distances are judged reasonably accurately for virtual distances around 1 m from the listener and are systematically underestimated as distance increases (Kolarik et al. 2013b, 2017a), similar to judgements of real sound sources (Mershon and Bowers 1979; Zahorik et al. 2005), suggesting that the simulation provided a reasonable reproduction of the acoustics of a real room.

As the stimuli were artificially generated, they may have lacked certain ecological cues. For example, the spectral coloration (i.e., modification of the sound spectrum) due to frequency-dependent absorption of the sound by the walls might be a reverberation-related distance cue (Bidart and Lavandier 2016), and the current simulation in which the reflections were not frequency dependent, would prevent the use of spectral coloration as a cue. However, Zahorik (2009) compared simplified model-based room simulations where only the direct-path and early reflections were spatially rendered with non-individualized HRTFs (similar to the current study) with simulations based on measurements from a real room. The results showed that spectral/timbre aspects of the stimuli made only a minimal contribution to estimated small room acoustics, a finding suggesting that the failure to simulate spectral coloration in the current study is likely to have had only a minimal effect.

### Procedures

Blindfolds were worn during the experiments by all participants. Participants had no prior knowledge of the real room in which they were tested. To prevent visual or auditory information regarding the testing room being available, participants were led into the room with blindfolds in place and wearing headphones. The experimenter instructed participants to imagine themselves sitting within a rectangular room of unspecified size in which loudspeakers would generate sounds at various distances, and participants were required to judge the distance of each sound in meters and centimeters, or feet if preferred. Participants were instructed that if the sound was perceived as being located within the head, they should report a distance of zero. No distances of zero were reported, so it can be assumed that participants perceived all sounds to be external to the head.

In a single block, the stimulus type (speech, music or noise) and condition (anechoic or reverberant) were fixed. Sounds at different distances were presented in a pseudo-random order. In each block, there were 80 trials with 10 repetitions of each virtual distance. After each presentation, the participant gave a verbal report of the apparent distance, with no time limitations for their response. Responses were recorded by the experimenter using a response interface. No training or feedback was given. The geometric mean of the 10 judgments for a given virtual distance was taken as the final estimate for that distance. After a block of trials was completed, participants gave judgments of the length, width, and height of the room for that block. There were 6 blocks [3 stimulus types (speech, music, or noise) and 2 room conditions (anechoic and reverberant)] and the order of presentation of the blocks was randomized (6 blocks, giving 480 trials in total). The experiment was completed in a single sitting of approximately 1 h and 40 min with as many breaks as required.

## Results

Data were analyzed using Pearson and Spearman correlations, mixed-model ANOVAs, and Kolmogorov–Smirnov normality tests.

For correlational analyses, in the majority of cases, the data were found to be normally distributed and Pearson correlations are reported. In cases where Kolmogorov–Smirnov tests indicated that the data did not follow a normal distribution, non-parametric Spearman correlations are reported, indicated by *s* in tables and plots.

Accuracy was assessed using a mixed-model ANOVA on the ratio of the judged distance to the simulated distance. To assess variability, a mixed-model ANOVA was run on the standard deviation (SD) of the ratio of the judged distance to the mean judged distance. Distance (three levels: near, middle and far), reverberation time (two levels: anechoic and reverberant rooms) and stimulus (three levels: speech, music and noise) were within-subjects factors and visual status (four levels: sighted, mild, mid-range, and severe VI) was a between-subject factor. For statistical analysis, data were grouped into near (1.22–1.72 m), middle (2.44–4.88 m), and far (6.90–13.79 m) distances. This procedure was similar to that used by Kolarik et al. (2020), which followed the methods utilized by Voss et al. (2015). Where sphericity was found to be violated, the Greenhouse–Geisser procedure was used to correct the degrees of freedom of the F-distribution. Behavioral data are available from OSF (https://osf.io/).

### Room volume estimates

**Fig. 2:**
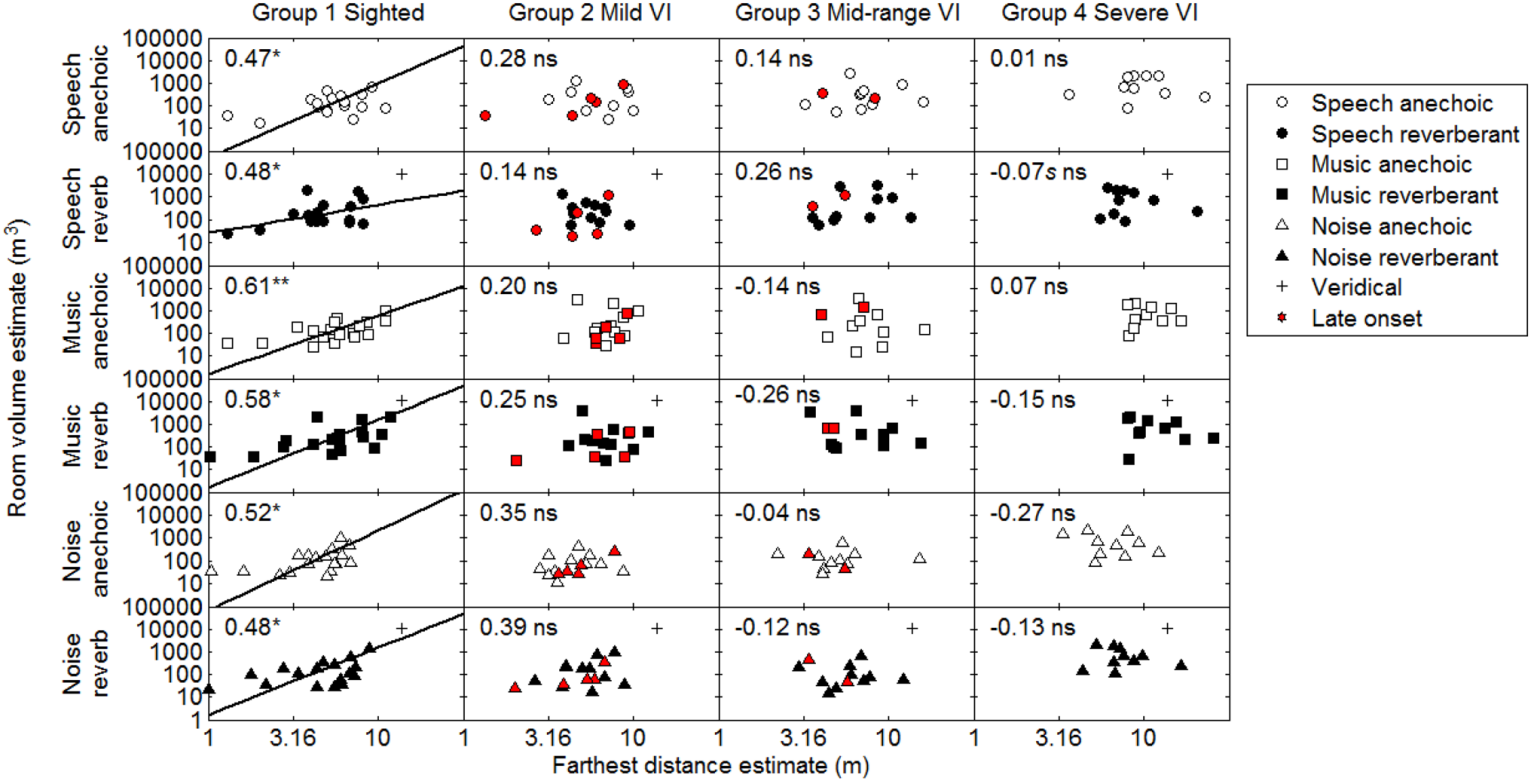
Room volume estimates plotted against farthest distance estimates. From left to right, the columns show results for groups 1–4. The rows show results for each condition. Pearson correlations between room size estimates and farthest distance estimates are shown in the upper left corner of each panel (**p < 0.01, *p < 0.05, ns: non-significant), and, where the correlation was significant, orthogonal fits to the data on log–log coordinates are shown by solid lines. *s* Indicates that a Spearman correlation is reported, where the data did not follow a normal distribution. Crosses for simulated reverberant rooms indicate veridical performance, for which the farthest distance was 13.8 m and the virtual room size was 10,500 m^3^. Data for late-onset VI participants are indicated by red symbols

Figure 2 shows individual room volume estimates plotted against farthest distance estimates, for each group. The latter were taken as the geometric mean of the 10 farthest virtual distance judgments for a given condition. Two estimates of participants from group 1 and one estimate from group 3 fell more than three standard deviations away from the overall mean within groups, and these points were considered as outliers and removed. In the reverberant virtual room, the majority of room volume and farthest distance judgments were underestimates, as the veridical simulated room size was 10,500 m^3^ and the farthest simulated sound distance was 13.8 m (shown by crosses in the panels of Fig. 2). The relationship between farthest distance estimates and room size judgments for each group was investigated using orthogonal regression and correlation, and *r* values are reported in the upper left corner of each panel of Fig. 2. Significant positive correlations (*p* < 0.05) between room size and farthest distance estimates were found for all stimuli and experimental conditions for the normally sighted group (group 1). No significant correlations were found for any of the VI groups.

### Room floor area estimates

**Fig. 3:**
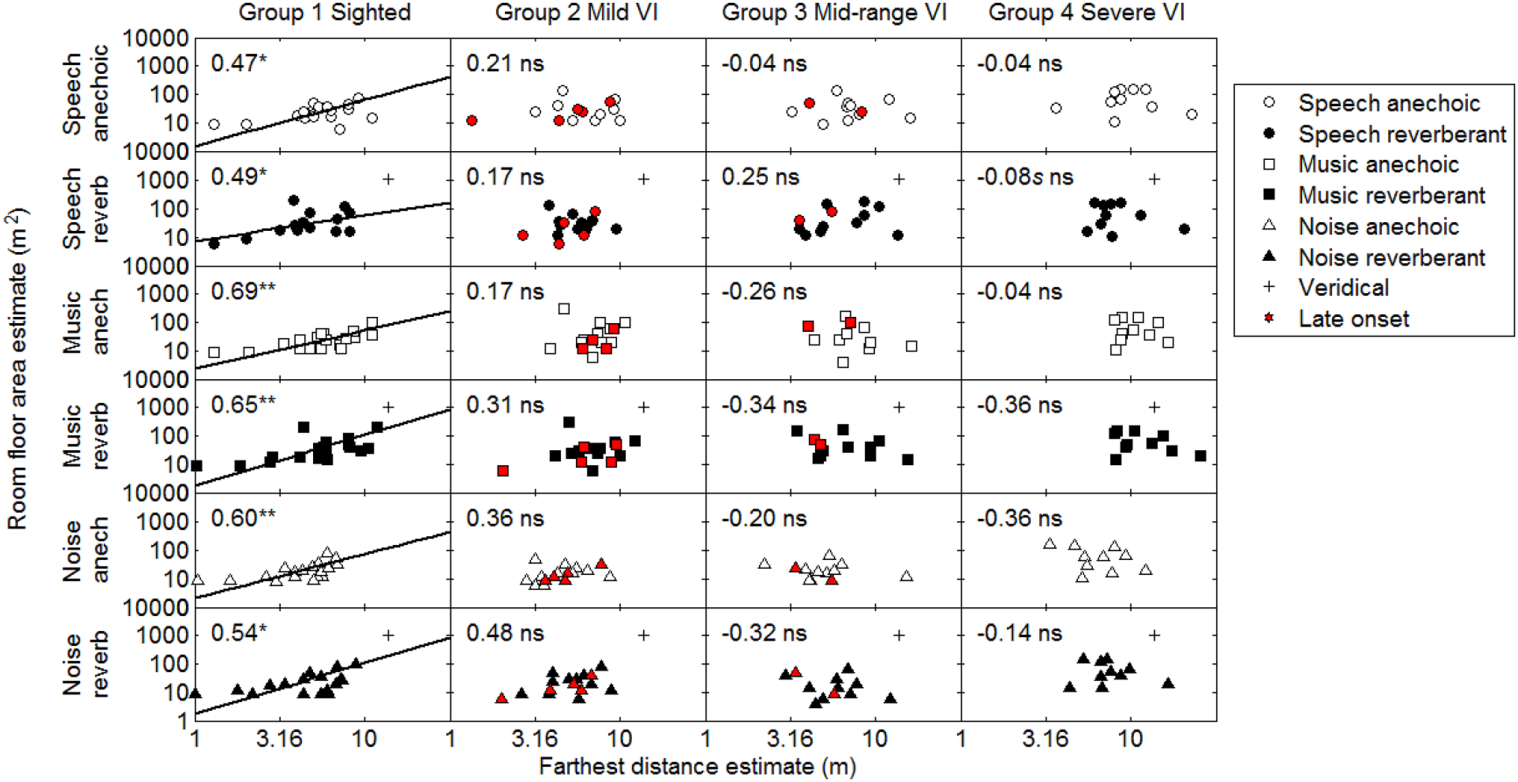
Symbols and panels as for Fig. 2, but for room floor area estimates plotted against farthest distance estimates. Crosses for simulated reverberant rooms indicate veridical performance, for which the farthest distance was 13.8 m, and the virtual floor area was 1050 m^2^

If the farthest judged sound source distance is used to infer room size, the farthest judged distance may be more highly correlated with room floor area than with volume estimates, as distance judgements provide no information about room height. Figure 3 shows individual room floor area estimates plotted against farthest distance estimates for each group. Similar to room volume estimates, the floor area in the reverberant virtual room was also usually underestimated. Significant positive correlations (*p* < 0.05) between room floor area estimates and farthest distance estimates were found for all conditions for the normally sighted participants (group 1). No significant correlations were found for any VI groups.

### Room dimension estimates

To examine the relationships between judgements for each of the room dimensions and farthest distance estimates, correlations were calculated between farthest distance estimates and judgements of room length, width and height for all stimuli, experimental conditions, and groups. No significant correlations were found for any of the VI groups for any of the stimuli and experimental conditions, with the exception of the width dimension in the reverberant noise condition for group 2 only (*p* < 0.05). For the sighted group, significant correlations were observed in almost all conditions for the length dimension and some conditions for the width dimensions, but not for the height dimension (see Table 2). These results are consistent with the idea that room size was inferred from the estimated distances of the farthest sources.

**Table 2:** Correlations between farthest estimated distances and estimated room length, width and height for the normally sighted control group (**p < 0.01, *p < 0.05)

Room dimension	Length	Width	Height
Speech anechoic	0.33*s*	0.26	0.32
Speech reverberant	0.56*	0.33	0.33
Music anechoic	0.64**	0.51*	0.32
Music reverberant	0.68**	0.50*	0.28
Noise anechoic	0.74**s	0.43	0.30
Noise reverberant	0.52*	0.51*	0.26

Pearson correlations were reported except for cases where where Kolmogorov–Smirnov tests indicated that the data did not follow a normal distribution, for which Spearman correlations are reported, denoted by *s*

### Room volume/floor area estimates and distance judgments, and accuracy and variability of the distance judgments

It is possible that relationships exist between room volume/floor area estimates and distance judgments other than farthest distance. To investigate other potential relationships, data were analyzed for room volume/floor area estimates and other distance estimates for all experimental conditions and groups. No significant correlations were found for any VI groups. For the sighted group, significant correlations were observed between room size and intermediate source distances in some conditions. However, no significant correlations were observed for the nearest source distance (see Table 3).

**Table 3:** Correlations between estimated distances and estimated room volume (upper panel) and estimated floor area (lower panel) for the four groups (**p < 0.01, *p < 0.05)

Estimated room volume									
Distance (m)	Group	1.22	1.72	2.44	3.45	4.88	6.90	9.75	13.79
Speech anechoic	1	0.33*s*	0.11*s*	0.20*s*	0.45*s*	0.32*s*	0.33*s*	0.29*s*	0.47*
	2	− 0.43	− 0.23	− 0.17	− 0.04	− 0.03	0.16	0.21	0.28
	3	− 0.44	− 0.48	− 0.30	− 0.08	− 0.12	0.05	0.08	0.14
	4	− 0.43	− 0.42	− 0.36	− 0.14	0.01*s*	− 0.20	− 0.14	0.01
Speech reverberant	1	0.08*s*	− 0.06*s*	0.15*s*	0.05*s*	0.24*s*	0.22*s*	0.47*	0.48*
	2	− 0.36	− 0.12	− 0.13	0.01*s*	− 0.14	− 0.06	− 0.02	0.14
	3	− 0.21	− 0.25	− 0.23	− 0.21	− 0.15	− 0.11	0.08	0.26
	4	− 0.26	− 0.21	− 0.29	− 0.27	− 0.20	− 0.12*s*	− 0.01	− 0.07*s*
Music anechoic	1	0.31	0.46	0.40*s*	0.54*	0.53*	0.57*	0.55*	0.61**
	2	0.03	0.03	− 0.04	− 0.05	− 0.02	0.11	0.07	0.20
	3	− 0.27	− 0.21	0.08	− 0.19	− 0.04	− 0.13	0.14	− 0.14
	4	− 0.37	− 0.33	− 0.26	− 0.12	− 0.01	0.05	0.15	0.07
Music reverberant	1	0.18	0.29	0.31*s*	0.48*	0.56*	0.57*	0.58*	0.58*
	2	− 0.19	− 0.16	0.00	0.07	0.16	0.25	0.27	0.25
	3	− 0.26	− 0.39	− 0.34	− 0.27	− 0.17	− 0.12	− 0.05	− 0.26
	4	− 0.46	− 0.41	− 0.39	− 0.41	− 0.22	− 0.25	− 0.03	− 0.15
Noise anechoic	1	0.39*s*	0.32	0.50*s	0.52*s	0.73**s	0.66**	0.59*	0.52*
	2	0.02	0.11	0.00	0.08	0.21	0.21	0.37	0.35
	3	− 0.14	− 0.02	0.16	0.20	0.11	0.30*s*	0.18	− 0.04
	4	− 0.43*s*	− 0.30	− 0.46*s*	− 0.39*s*	− 0.02*s*	− 0.41	− 0.28	− 0.27
Noise reverberant	1	0.34*s*	0.55*	0.60*s	0.46*s*	0.66**s	0.59*	0.49*s	0.48*
	2	0.10	0.10	0.15	0.22	0.23	0.29	0.30	0.39
	3	0.06	0.14	0.23	0.25	0.16	0.22	0.10	− 0.12
	4	− 0.21*s*	− 0.29	− 0.21	− 0.16	− 0.18	− 0.17	− 0.21	− 0.13
Estimated floor area									
Distance (m)		1.22	1.72	2.44	3.45	4.88	6.90	9.75	13.79
Speech Anechoic	1	0.14*s*	0.01*s*	0.22*s*	0.50*s	0.25*s*	0.35*s*	0.31*s*	0.47*
	2	− 0.40	− 0.27	− 0.23	− 0.10	− 0.08	0.07	0.15	0.21
	3	− 0.45	− 0.50	− 0.37	− 0.19	− 0.25	− 0.12	− 0.09	− 0.04
	4	− 0.49	− 0.47	− 0.43	− 0.21	− 0.05*s*	− 0.27	− 0.19	− 0.04
Speech reverberant	1	− 0.07*s*	− 0.07*s*	0.15*s*	− 0.02*s*	0.20*s*	0.28*s*	0.47*	0.49*
	2	− 0.24	− 0.04	− 0.07	0.05*s*	− 0.09	− 0.02	0.00	0.17
	3	− 0.16	− 0.17	− 0.16	− 0.12	− 0.09	− 0.11	0.09	0.25
	4	− 0.31	− 0.26	− 0.35	− 0.34	− 0.30	− 0.10*s*	− 0.08	− 0.08*s*
Music anechoic	1	0.41	0.53*	0.48*s	0.61*	0.60**	0.62**	0.64**	0.69**
	2	0.06	0.05	0.00	− 0.02	− 0.02	0.13	0.06	0.17
	3	− 0.32	− 0.24	0.03	− 0.25	− 0.14	− 0.20	− 0.01	− 0.26
	4	− 0.37	− 0.34	− 0.34	− 0.23	− 0.09	− 0.03	0.07	− 0.04
Music reverberant	1	0.23	0.33	0.38*s*	0.52*	0.60**	0.61**	0.64**	0.65**
	2	− 0.21	− 0.16	0.01	0.11	0.21	0.28	0.31	0.31
	3	− 0.31	− 0.41	− 0.42	− 0.37	− 0.26	− 0.18	− 0.15	− 0.34
	4	− 0.57	− 0.53	− 0.55	− 0.58	− 0.41	− 0.44	− 0.27	− 0.36
Noise anechoic	1	0.27*s*	0.38	0.49*s	0.49*s	0.71**s	0.66**	0.63**	0.60**
	2	0.03	0.09	− 0.03	0.08	0.19	0.15	0.31	0.36
	3	− 0.17	− 0.03	0.16	0.15	− 0.04	0.35*s*	0.02	− 0.20
	4	− 0.48*s*	− 0.38	− 0.42*s*	− 0.60*s*	− 0.10*s*	− 0.49	− 0.38	− 0.36
Noise reverberant	1	0.18*s*	0.44	0.54*s	0.37*s*	0.65**s	0.58*	0.54*s	0.54*
	2	0.07	0.07	0.15	0.22	0.28	0.34	0.39	0.48
	3	0.13	0.18	0.27	0.24	0.07	0.10	− 0.04	− 0.32
	4	− 0.36*s*	− 0.34	− 0.28	− 0.23	− 0.22	− 0.20	− 0.23	− 0.14

Pearson correlations were reported except where the data did not follow a normal distribution, for which Spearman correlations are reported, indicated by *s*

**Fig. 4:**
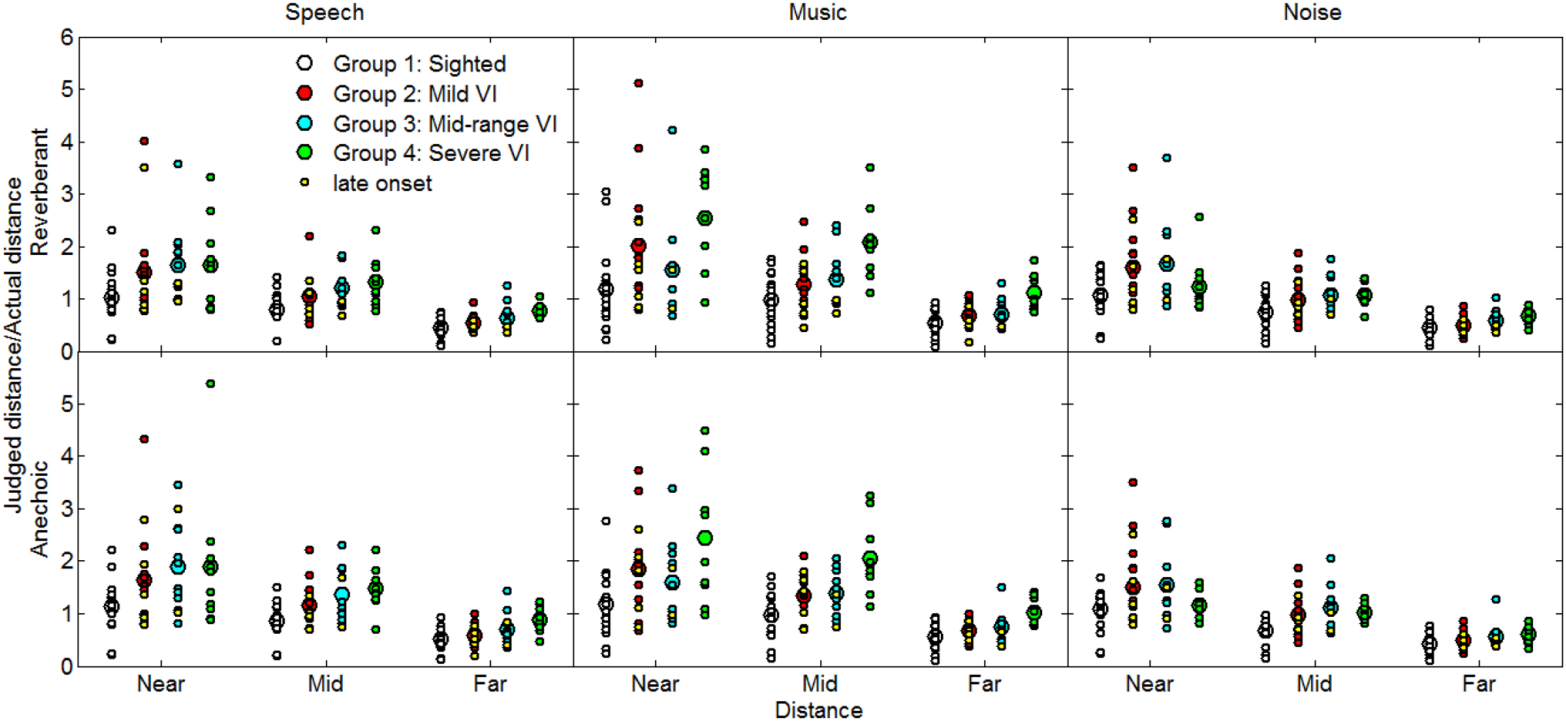
Ratio of judged to simulated distances (a measure of accuracy) for normally sighted (white circles), mild VI (red circles), mid-range VI (blue circles) and severe VI (green circles) groups. Large symbols show mean data, and small symbols show individual data. Late-onset VI participants are indicated by small yellow circles. The bottom and top rows show data for the virtual anechoic and reverberant rooms, respectively. The left, middle and right columns show data for speech, music and noise, respectively

To assess the accuracy of the distance judgments, for each participant, condition and simulated distance, the ratio of the judged distance to the simulated distance was calculated. The mean ratios for the normally sighted group and VI groups are shown in Fig. 4. All groups made systematic errors, the ratios decreasing with increasing virtual distance. The finding that the sighted group were on average approximately accurate for small distances and systematically underestimated the judged distance as the simulated distance increased, is consistent with previous reports using both real and simulated environments (Mershon and Bowers 1979; Loomis et al. 1998; Zahorik and Wightman 2001; Zahorik 2002a), see Zahorik et al. (2005) and Kolarik et al. (2016a) for reviews. These findings suggest that the virtualization methods used in the current study provided an adequate simulation of auditory distance. Overall, the judged distances were greater for the VI groups than for the sighted group, with the VI groups being less accurate for shorter distances and more accurate for large distances. These findings contrast with previous work with fully blind participants, who were generally not more accurate for farther distances (compare Figs. 3–4 of Kolarik et al. (2017a). Possible reasons for the discrepancy are discussed later in this paper. A mixed-model ANOVA on the ratio of the judged distance to the simulated distance showed main effects of visual status (*F*(3, 52) = 5.5, *p* = 0.002, *η*^2^ = 0.24), distance (*F*(1.05, 54.64) = 122.4, *p* = 0.001, *η*^2^ = 0.70), and stimulus (*F*(2, 104) = 43.2, *p* = 0.001, *η*^2^ = 0.45), and significant interactions between stimulus and visual status (*F*(6, 104) = 9.0, *p* = 0.001, *η*^2^ = 0.34), stimulus and distance (*F*(2.36, 122.48) = 6.6, *p* = 0.001, *η*^2^ = 0.11), stimulus and reverberation time (*F*(2, 104) = 9.2, *p* = 0.001, *η*^2^ = 0.15), and stimulus, distance and visual status (*F*(7.07, 122.48) = 4.46, *p* = 0.001, *η*^2^ = 0.21). No other significant main effects or interactions were observed (all *p* > 0.05). Post hoc *t*-tests with Bonferroni correction showed significant differences between the the sighted group and the severe VI group for middle and far distances for the speech stimuli, and for all distances for the music stimuli. Significant differences between the sighted group, and the mid-range and severe VI groups were observed for anechoic noise stimuli at middle distances.

Table 4 shows correlations between simulated distances and estimated distances for each group. With the exception of the anechoic music condition, for which the correlations were similar across groups, correlations generally decreased as the severity of visual loss increased. The effects of the severity of VI on auditory estimates of distance and room size are analyzed and described in another paper from our laboratory (Kolarik et al. 2020).

**Table 4:** Correlations between simulated distances and estimated distances for each group (**p < 0.01)

Group	1	2	3	4
Speech anechoic	0.66**	0.57**	0.57**	0.45**
Speech reverberant	0.65**	0.61**	0.59**	0.43**
Music anechoic	0.63**	0.69**	0.63**	0.69**
Music reverberant	0.58**	0.58**	0.58**	0.43**
Noise anechoic	0.63**	0.57**	0.52**	0.56**
Noise reverberant	0.65**	0.58**	0.63**	0.52**

**Fig. 5:**
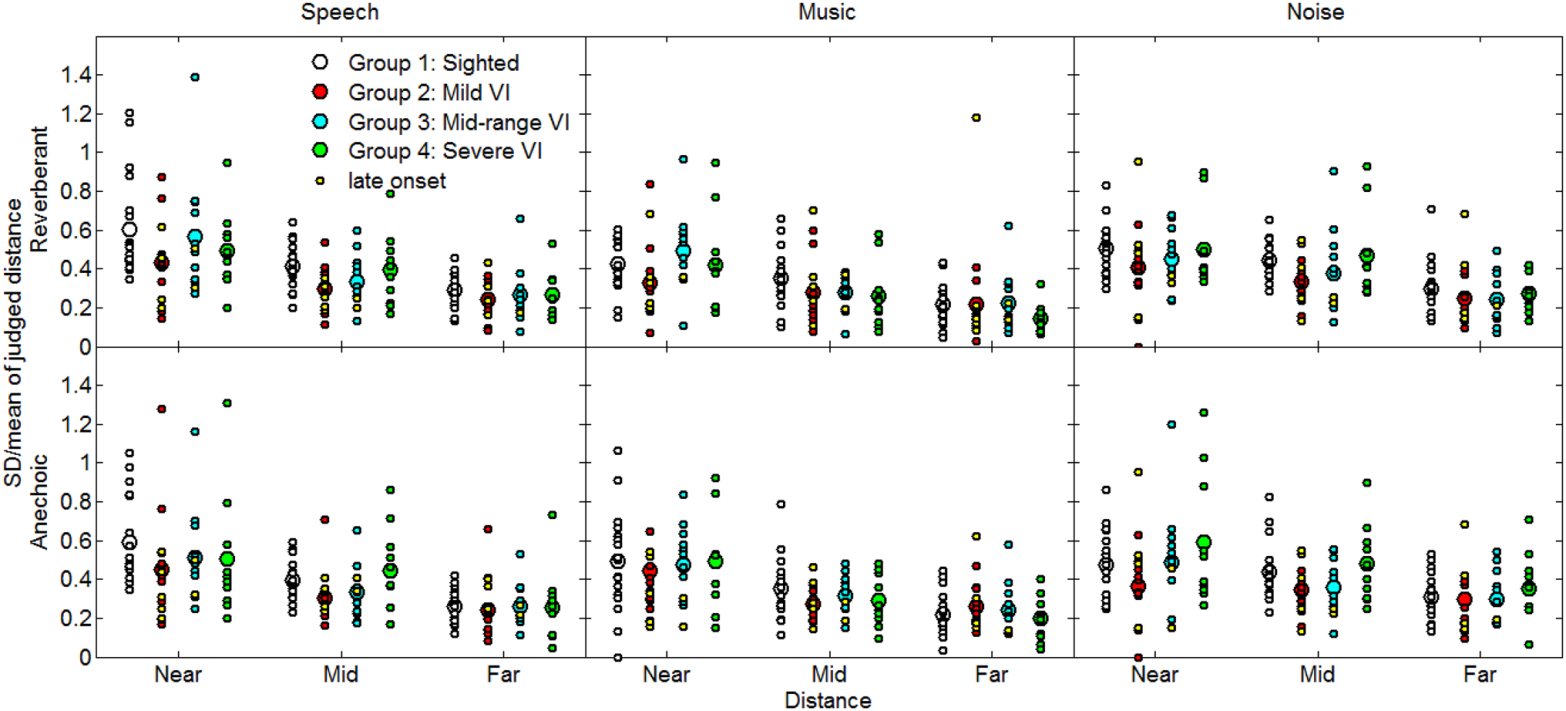
SD of the ratio of judged distance to mean judged distance (a measure of variability) for normally sighted and VI groups. Otherwise as Fig. 4

To assess the variability of the distance judgments, for each participant, condition and simulated distance we determined the standard deviation (SD) of the ratio of the judged distance to the mean judged distance. The mean and individual ratios are shown in Fig. 5. In general, variability decreased as distance increased in all conditions, and was approximately similar for the sighted and VI participants. A mixed-model ANOVA showed significant main effects of stimulus (*F*(2, 104) = 9.8, *p* = 0.001, *η*^2^ = 0.16) and distance (*F*(1.38, 71.77) = 112.3, *p* = 0.001, *η*^2^ = 0.68), and a significant interaction between stimulus and distance (*F*(2.93, 152.46) = 4.0, *p* = 0.009, *η*^2^ = 0.07). No other significant main effects or interactions were observed (all *p* > 0.05) including visual status, indicating no significant differences in the variability of the distance judgments between sighted controls and VI participants.

To investigate whether age of visual loss onset affected performance, additional analyses were run on data for early-onset VI participants only, so that five late-onset participants were excluded from group 2 and two from group 3 (see Table 5). There were no significant positive correlations between room size and farthest distance estimates, or between room floor area estimates and farthest distance estimates, for any stimuli and and experimental conditions for VI groups 2 and 3 (*p* > 0.05). A mixed-model ANOVA on the accuracy of distance judgements showed main effects of visual status (*F*(3, 45) = 5.6, *p* = 0.002, *η*^2^ = 0.27), distance (*F*(1.05, 47.25) = 109.8, *p* = 0.001, *η*^2^ = 0.71), and stimulus (*F*(2, 90) = 39.3, *p* = 0.001, *η*^2^ = 0.47), and significant interactions between stimulus and visual status (*F*(6, 90) = 8.2, *p* = 0.001, *η*^2^ = 0.35), stimulus and distance (*F*(2.4, 108.08) = 6.9, *p* = 0.001, *η*^2^ = 0.13), stimulus and reverberation time (*F*(2, 90) = 11.1, *p* = 0.001, *η*^2^ = 0.2), and stimulus, distance and visual status (*F*(7.21, 108.08) = 4.98, *p* = 0.001, *η*^2^ = 0.25). No other significant main effects or interactions were observed (all *p* > 0.05). A mixed-model ANOVA on the variability of the distance judgments showed significant main effects of stimulus (*F*(2, 90) = 8.9, *p* = 0.001, *η*^2^ = 0.17) and distance (*F*(1.35, 60.65) = 118.4, *p* = 0.001, *η*^2^ = 0.76), and a significant interaction between stimulus and distance (*F*(3.04, 136.78) = 4.7, *p* = 0.004, *η*^2^ = 0.09). No other significant main effects or interactions were observed (all *p* > 0.05). These results show that similar findings were observed whether or not late-onset VI participants were included in the analyses, suggesting that age of onset of VI did not affect the results.

Although the experiment was designed to minimize auditory and visual information about the testing room, as described earlier, it is possible that other clues about the actual testing room contributed to the judgments of room size. The volume of the testing room was 22.5 m^3^ and the floor area was 9 m^2^, both considerably smaller than for the simulated room, which had a volume of 10,500 m^3^, and a floor area of 1050 m^2^. Had the dimensions of the testing room affected the judgments, then the estimated size for the virtual room would have been of similar size as the testing room. In fact, the estimates of volume, room size, and room length were markedly larger than the dimensions of the testing room, suggesting very little influence when making spatial judgments.

In summary, the patterns of correlations between farthest distance judgments and judged room volume and between farthest distance judgments and judged room floor area were similar (Figs. 2 and 3). For the sighted participants, significant positive correlations were observed for all conditions. For the groups with VI, no significant correlations were found in any condition.

## Discussion

The main findings of this study are:

1. For participants with VI, there were no significant correlations between acoustically judged room size and judged farthest sound source distance, whereas for sighted participants significant correlations were found. These findings are consistent with the perceptual deficiency hypothesis, suggesting that the use of a degraded visual signal to calibrate audition can interfere with the relationship between the estimated farthest sound distance and the room size.
2. Even mild VI was associated with no significant correlation between judged room size and judged farthest sound source distance.

When both auditory and visual information are available to normally sighted participants, judgments of room size are primarily based on visual information (Maempel and Jentsch 2013). Calibration of auditory space is, thus, assumed to be based on visual spatial information, which is far more accurate than auditory spatial information (Da Silva 1985; Loomis et al. 1998). It has been suggested that the judged distance of the farthest sound source can be used to estimate the minimum possible distance to the far wall, as hypothesized by Cabrera et al. (2005) and Calcagno et al. (2012), who suggested that auditory judgements of distance and room size should be related. Kolarik et al. (2013d), showed that these variables were indeed positively correlated for sighted participants (*r* = 0.46). Our current data support this hypothesis. The strategy of using farthest distance estimates to judge room size is particularly useful when first entering an unfamiliar room to gain insights regarding its dimensions. Although such a strategy results in a conservative estimate of room size unless the farthest sound source is close to the far wall, it at least allows an initial internal representation to be formed to support path planning and navigation that can be updated later when further spatial cues become available.

It is likely that participants with VI rely on sources of information other than farthest distance to estimate the size of the room. For example, for reverberant rooms, participants with VI may base their judgments of distance on the ratio of direct to reverberant sound energy, while their judgments of room size may be based on the overall perceived amount or duration of the reverberation (Sandvad 1999), a possibility that is beyond the scope of the current study and needs further investigation. The finding that the sound source distance and room size judgments did not differ markedly for anechoic and reverberant simulated rooms may indicate that participants with VI largely based their distance judgments on the sound level at their ears (which decreased with increasing simulated distance) and that they simply guessed a reasonable number when judging room size. The similarity of the judgments for the simulated reverberant and anechoic rooms may have been due to the relatively short room reverberation time (*T*_60_ = 700 ms) used. Longer reverberation times might provide more salient cues for room size. Previous studies using reverberation times of 1700 ms or more have shown that rooms with long reverberation times are judged to be larger than rooms with short reverberation times (Mershon et al. 1989; Etchemendy et al. 2017).

Previous work from our laboratory showed that for fully blind participants, for whom visual calibration information was completely absent, there was a stronger correlation (*r* = 0.83) between auditory judgments of farthest distance and room size than observed for sighted controls (*r* = 0.46), a finding consistent with the perceptual enhancement hypothesis (Kolarik et al. 2013d). It is perhaps surprising that there was a strong correlation for participants who were fully blind, but not for VI participants tested here for whom the results were consistent with the perceptual deficiency hypothesis. Differences between participants with full blindness and severe VI have been reported previously. Lessard et al. (1998) showed that fully blind participants were more accurate at localizing sounds in azimuth than sighted controls, but participants with VI were less accurate than either the fully blind or sighted groups. The authors suggested several possible explanations for these findings: (1) VI participants demonstrated abnormal orienting behaviors that might have interfered with their judgments of sound azimuth; (2) the development of an internal auditory spatial map partly based on residual vision and partly based on other information might have affected the accuracy of the overall map; and (3) auditory compensation from recruitment of deafferented sensory brain areas (in line with perceptual enhancement hypothesis) may have occurred for the fully blind participants, but not for the VI participants because of their remaining vision. These explanations would also apply to the current findings for judgments of auditory distance and room size. It is also possible that since blind participants cannot use any visual calibration information regarding room size, and also cannot use sensorimotor contingencies (O'Regan and Noë 2001) over large distances to calibrate auditory room size judgments, they are likely to depend more strongly than sighted participants on utilizing the judged distance of the farthest sound source to infer the nearest possible distance of the far wall, leading to the strong correlation reported by Kolarik et al. (2013d).

Although the current findings and those of previous work that tested VI participants (Lessard et al. 1998) support the perceptual deficit hypothesis, other studies have reported that VI participants show significantly better azimuth localization abilities than sighted participants (Dufour and Gérard 2000; Després et al. 2005a), consistent with the perceptual enhancement hypothesis. Cappagli et al. (2017) also showed that VI children performed significantly better than sighted children when performing a dynamic task involving touching the perceived endpoint of a moving sound. The differences in findings across studies may be due to differences in the visual status of the participants tested and task differences. For example, the current study tested participants with a range of eye conditions using distance and room size judgment tasks, contrasting with previous work that tested myopic participants only for azimuth localization (Dufour and Gérard 2000; Després et al. 2005a). The differences across studies suggest that the effects of VI on auditory abilities are task specific, as is the case for full blindness (Voss 2016). Future studies are required to clarify which auditory abilities are enhanced by VI and which are degraded, and to investigate how the type of visual loss affects auditory abilities.

In the current experiment, participants reported estimates in cm, m or feet if they preferred. If VI participants did not use such units in their daily lives, they might have inaccurate internal estimates of what constitutes cm, m or feet. However, discussion with the VI participants indicated that they did use cm, m or feet to report distances in their daily lives, as do sighted individuals. We previously conducted a control experiment with fully blind individuals to check that their perception of what a meter constitutes does not differ significantly from that for sighted controls (Kolarik et al. 2017a). In this experiment, groups of sighted and blind individuals were blindfolded and asked to walk predetermined distances ranging from 2 to 10 m. No significant differences between groups were observed. However, the participants in the current study gave verbal estimates, and it is possible that a different method of reporting, such as walking to the judged location of the sound source, might lead to more veridical judgments. Further studies using different response methods would be needed to investigate this.

Changes in auditory localization abilities in fully blind individuals have been linked to plasticity of the occipital cortex that occurs during a critical period (Cohen et al. 1999). Consistent with this, early-onset blind participants often display enhanced localization abilities compared to late-onset blind (Collignon et al. 2009). In the current study, there was no clear indication that early-onset VI participants performed differently to late-onset VI participants. Whether or not a critical period for plasticity of the occipital cortex exists for VI individuals is not known, and future work is needed to investigate whether early-onset VI participants display different auditory abilities to late-onset VI participants.

The present results add to the growing body of evidence that full blindness is not necessary for significant changes in hearing to become manifest. The World Health Organization estimates that across the world 188.5 million people have mild visual loss, 217 million have moderate to severe losses, and 36 million have full blindness (Bourne et al. 2017). Given that loss of sight results in increased reliance on sound for communication, socialization, alerts of danger, and spatial awareness, further work is needed to establish how different auditory abilities are affected by partial as well as full visual loss, and the effect of these changes in auditory abilities on daily activities.

In conclusion, the findings show that partial visual loss disrupts the relationship between judged room size and sound source distance that is evident in participants with full vision. Consistent with the perceptual deficiency hypothesis, the results support the idea that using a degraded visual signal to calibrate auditory space interferes with the relationship between the estimated room size, and the farthest sound distance.

## Appendix

See Table 5.

**Table 5:** Details of the visually impaired participants

Cause of visual loss, VI group	Age (years)	Better eye visual acuity (LogMAR)	Age of onset of visual loss (years)	Early (E) or late (L) onset	Duration of visual loss (years)
Hypoplastic Disc, 2	25	0.40	Birth	E	25
Retinitis Pigmentosa, 2	20	0.40	12	L	8
Neuritis, 2	23	0.40	1	E	22
Stargardt's, 2	24	0.50	6	E	18
Bietti's crystalline dystrophy, 2	31	0.50	4 months	E	31
Retinitis Pigmentosa, 2	21	0.60	Birth	E	21
Retinitis Pigmentosa, 2	19	0.60	17	L	2
Retinitis Pigmentosa, 2	20	0.60	Birth	E	20
Marfan Syndrome, 2	20	0.70	10	L	10
Cone rod dystrophy, 2	18	0.70	Birth	E	18
Nystagmus, 2	20	0.90	Birth	E	20
Amblyopia, 2	18	0.90	Birth	E	18
Retinitis Pigmentosa, 2	25	0.90	14	L	11
Stargardt's, 2	21	1.00	Birth	E	21
Cone dystrophy, 2	18	1.00	10	L	8
Hypoplastic Disc, 2	24	1.00	Birth	E	24
Retinitis Pigmentosa, 3	18	1.10	Birth	E	18
Stargardt's, 3	23	1.10	Birth	E	23
Retinitis Pigmentosa, 3	18	1.10	11	L	7
Healed retinitis, 3	23	1.10	Birth	E	23
Stargardt's, 3	20	1.28	17	L	3
Retinitis Pigmentosa, 3	20	1.28	4	E	16
Cone rod dystrophy, 3	18	1.28	Birth	E	18
Leber congenital amaurosis, 3	22	1.28	Birth	E	22
Stargardt's, 3	27	1.48	Birth	E	27
Retinitis Pigmentosa, 3	28	1.77	6	E	22
Pale disc, 3	17	2.00	Birth	E	17
Pale disc, 3	19	2.30	1.5	E	17.5
Retinitis Pigmentosa, 4	19	3.00	Birth	E	19
Optic atrophy, 4	26	3.00	3	E	23
Optic atrophy, 4	18	3.00	Birth	E	18
Glaucoma, 4	31	3.00	Birth	E	31
Retinal detachment, 4	20	3.00	4	E	16
Glaucoma, Aphakia, Pseudophakia, 4	18	3.00	Birth	E	18
Retinitis Pigmentosa, 4	22	3.50	5	E	17
Phthisical eye, 4	19	3.50	Birth	E	19
Corneal opacity, 4	22	3.50	Birth	E	22
Total corneal opacity, 4	24	4.00	Birth	E	24

The cause of VI, age, age of onset of VI, early (E) or late (L) onset VI, duration of VI, and better eye visual acuity are given for each participant. Groups 2, 3 and 4 had mild, mid-range, and severe VI, respectively

## Declarations

### Availability of data and material

The datasets generated during and/or analyzed during the current study are available from the corresponding author on reasonable request.
